# Dynamic X-ray Coherent Diffraction Analysis: Bridging the Time Scales between Imaging and Photon Correlation Spectroscopy

**DOI:** 10.1021/acs.nanolett.4c03699

**Published:** 2024-10-18

**Authors:** Gerard N. Hinsley, Fabian Westermeier, Bihan Wang, Kuan Hoon Ngoi, Shweta Singh, Rustam Rysov, Michael Sprung, Cameron M. Kewish, Grant A. van Riessen, Ivan A. Vartanyants

**Affiliations:** † Deutsches Elektronen-Synchrotron DESY, Notkestr. 85, Hamburg 22607, Germany; ‡ Center for Transformative Science, Shanghai Technical University, Shanghai 201210, China; ¶ Australian Nuclear Science and Technology Organisation, Australian Synchrotron, Victoria 3168, Australia; § Department of Mathematical and Physical Sciences, La Trobe University, Bundoora, Victoria 3086, Australia; ∥ Melbourne Centre for Nanofabrication, Clayton, Victoria 3168, Australia

**Keywords:** coherent X-ray diffractive imaging, X-ray photon correlation spectroscopy, dynamics, nanoparticles

## Abstract

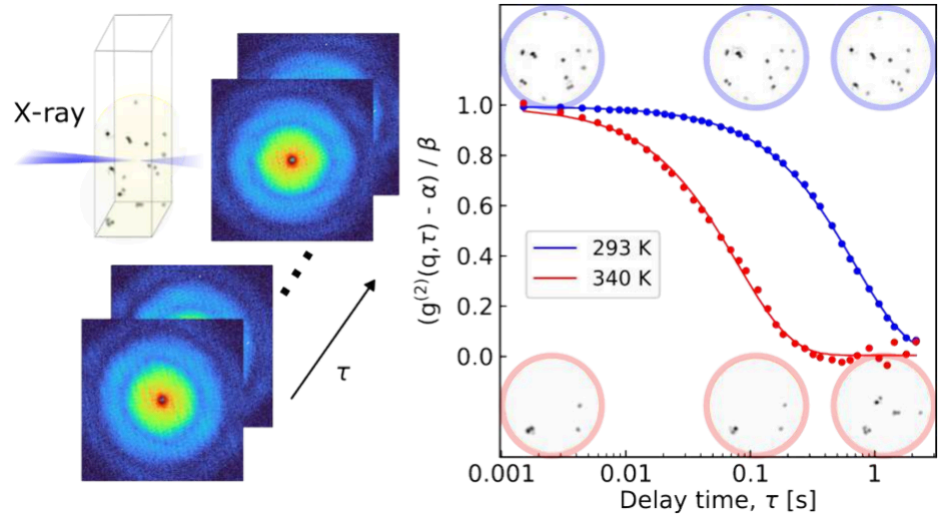

The advent of diffraction limited sources and developments in detector technology opens up new possibilities for the study of materials *in situ* and *operando*. Coherent X-ray diffraction techniques such as coherent X-ray diffractive imaging (CXDI) and X-ray photon correlation spectroscopy (XPCS) are capable for this purpose and provide complementary information, although due to signal-to-noise requirements, their simultaneous demonstration has been limited. Here, we demonstrate a strategy for the simultaneous use of CXDI and XPCS to study *in situ* the Brownian motion of colloidal gold nanoparticles of 200 nm diameter suspended in a glycerol–water mixture. We visualize the process of agglomeration, examine the spatiotemporal space accessible with the combination of techniques, and demonstrate CXDI with 22 ms temporal resolution.

Understanding the behavior and function of materials at the nanoscale necessitates the use of *in situ* and *operando* characterization approaches. Techniques using coherent X-ray illumination are becoming increasingly capable for the purpose of characterizing nanostructures *in situ* with diffraction limited sources^1,2^ and the development of new fast-framing detectors.^3,4^

Two complementary techniques, X-ray photon correlation spectroscopy (XPCS)^5,6^ and coherent X-ray diffraction imaging (CXDI),^7^ can take advantage of these improvements for the analysis of sequentially measured coherent diffraction patterns. XPCS analyzes the correlation in the measured intensities *I*(*q*, *t*) at time *t* and after some delay τ for a given scattering vector *q*, and has been widely used to study *in situ* and *operando* dynamics.^8−12^ The information obtained by XPCS, however, is the average over the entire illuminated region, and as such, is insensitive to minute changes that occur on an individual basis. This limitation may be overcome by the use of imaging techniques which are able to provide real-space structural information, allowing localized dynamics to be observed.

CXDI, alternatively, is a lensless imaging method which is able to provide real-space images of an object with nanoscale spatial resolution.^7^ To achieve this, CXDI requires the measurement of oversampled far-field diffraction patterns as input into phase-retrieval algorithms.^13^ These algorithms provide an estimate of the missing phase information on the wavefield at the detector plane, which through the Fourier transform relationship between the detector and object planes, can then provide images of the original object. The resolution of this approach is, in principle, limited by the largest angle at which the scattered intensity is recorded on the detector. The inversion process of these diffraction patterns, however, is quite difficult, typically requiring a significantly higher signal-to-noise ratio (SNR) than XPCS for the algorithms to converge to an accurate solution. This limitation has driven interest in the development of novel strategies to improve the reliability of reconstructions. One of these methods, ptychography, introduces translational diversity in a set of diffraction patterns, where the overlap between adjacent frames can be exploited as a strong constraint allowing many experimental constraints to be relaxed. Consequently, ptychography has been attractive to use for the study of many systems *in situ*,^14−18^ however this requirement of a high degree of spatial overlap introduces a trade-off between temporal resolution and the scanning area. As such, alternative strategies have been developed to improve the robustness of CXDI for imaging dynamic systems.^19−23^ This has resulted in the first demonstration of the complementary information obtained by XPCS and CXDI which reached a temporal resolution of 0.1 s^24^ — an order of magnitude faster than the 1.2 s achieved using ptychography.^25^ The experimental arrangement used in this high temporal resolution CXDI demonstration, however, required the use of a triangular aperture and the *a priori* characterization of the probe function through a ptychography measurement.

Here, we demonstrate how these results could be improved through a combination of XPCS and CXDI data analysis methods applied to an extended time-series acquisition of high frame-rate data. By first reconstructing data integrated over many diffraction patterns, we obtain images of a blurred object with corresponding support functions. The CXDI reconstructions are then refined by using these supports as an initial guess for the reconstruction of smaller subsets of data, resulting in clearer images with high temporal resolution. This approach then allows for the simultaneous application of CXDI and XPCS while requiring no reference aperture or knowledge of the probe beam.

We use this approach to study the Brownian motion of Au colloidal nanoparticles suspended in a dilute glycerol–water mixture inside a thin capillary. We demonstrate how performing these measurements simultaneously provides complementary information on the nature of the dynamics, while also demonstrating CXDI with a temporal resolution of 22 ms.

The samples consist of spherical nanoparticles of diameter 200 nm with a 3% polydispersity, which are covered with a 3 kDa poly(ethylene glycol) methyl (PEG) ligand. The dilute solution (0.002% volume fraction) was placed into a rectangular capillary with dimensions 0.5 mm × 5 mm × 0.05 mm (*H* × *V* × *W*), where the width (W) was aligned perpendicular to the axis of the beam direction. Data were collected at the P10 Coherence Applications beamline at PETRA III, where X-rays of 8 keV photon energy were focused by compound refractive lenses to a size of 2.8 × 2.3 μm^2^ (*H* × *V*) at the sample, and the incident flux was approximately 9.6 × 10^10^ photons per second. We collected 21,000 coherent diffraction patterns using an EIGER 4M detector at a rate of 714 Hz (total acquisition time ≈ 30 s), which was placed 5 m downstream of the sample position. We studied the dynamics at two different temperatures, 293 and 340 K, in order to examine the accessible spatiotemporal space by CXDI. In-depth experimental details are provided in the Supporting Information (SI).

To analyze the data using XPCS, the normalized intensity autocorrelation function, *g*^(2)^(*q*, τ), is calculated by
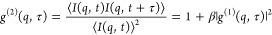
1where β is the speckle contrast, and *g*^(1)^(*q*, τ) is the intermediate scattering function. For a system exhibiting Brownian motion, *g*^(1)^(*q*, τ) can be described by^6^
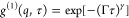
2where Γ = *Dq*^2^ is the relaxation rate with *D* being the diffusion coefficient of the particles, and γ is a measure of the distribution of relaxation times. We fit the XPCS data using
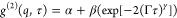
3where α represents the baseline, and we set γ = 1 as a fixed parameter as the motion of the nanoparticles is expected to be Brownian. Fits with γ as a free parameter are shown in the SI.

Figure 1a,b presents the results of this analysis performed over 3 s near the beginning of each data set at temperatures of 293 and 340 K, respectively. The solid points represent values of *g*^(2)^(*q*, τ) calculated by eq 1, and were normalized by subtracting the baseline and division by the contrast value obtained from the fits. The solid lines in Figure 1a,b are the fits for each *q* partition obtained by eq 3. These results clearly indicate the expected behavior where the effect of increasing the temperature of the system translates to a decay in the correlation at shorter delay times. The XPCS results for the whole time series, as well as further details of the fitting procedure, are included in the SI.

**Figure 1:**
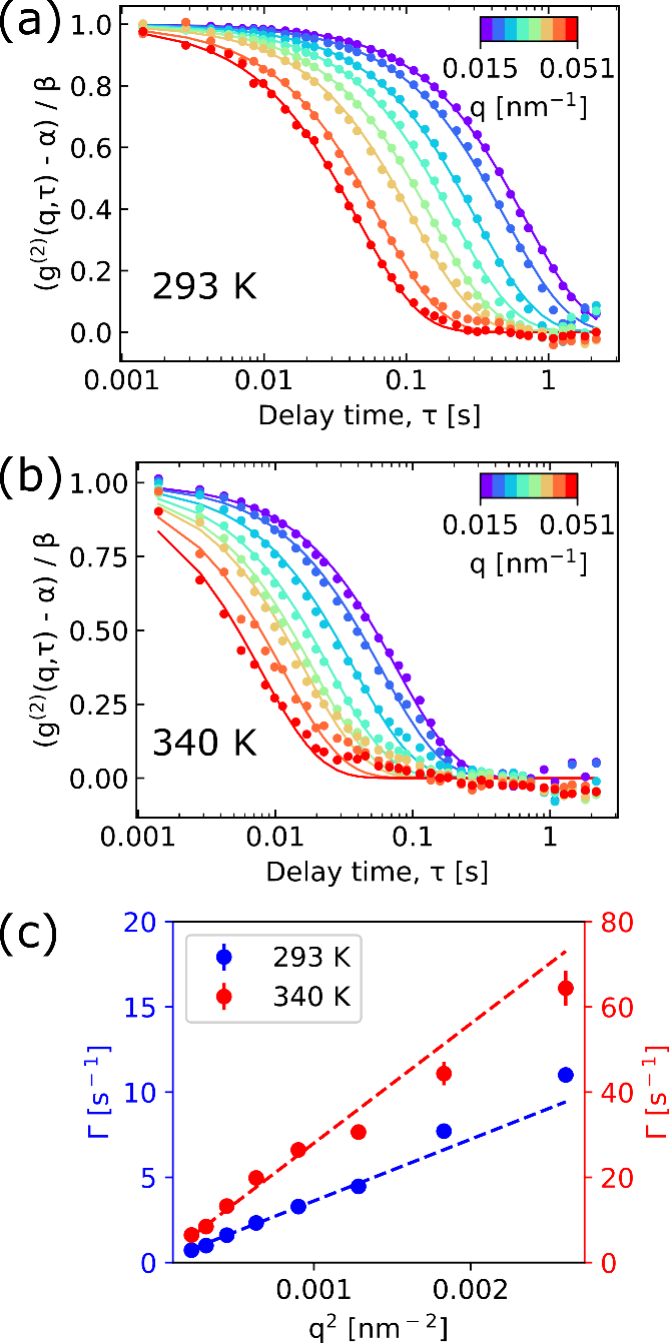
Experimental *g*^(2)^(*q*, τ) functions (points) and their fits (solid lines) as a function of delay time at temperatures of 293 K (a) and 340 K (b) over 3 s of data near the beginning of the series. The color represents the eight different *q*-partitions used for the analysis, where the partitions are spaced with equal d*q*/*q* steps. (c) Plot of Γ as a function of *q*^2^ for both temperatures. Linear fits are indicated by the dashed lines, and provide values of: *D*_293_ = 3,618 ± 18 nm^2^/s and *D*_340_ = 28,024 ± 423 nm^2^/s.

Figure 1c shows a plot of the values of Γ obtained from the fitting procedure obtained at both temperatures. By fitting the results in Figure 1c, we obtain estimates of the diffusion coefficient of *D*_293_^XPCS^ = 3,618 ± 18 nm^2^/s and *D*_340_^XPCS^ = 28,024 ± 423 nm^2^/s. With the results obtained at 293 K, we perform a microrheology analysis (details in the SI) and estimate that the solution contains approximately 88% glycerol and 12% water. It follows that for a solution with this composition at a temperature of 340 K, we expect that *D* = 78,388 nm^2^/s. This value is about three times the actual value obtained, indicating that our nanoparticles are less mobile than expected. This unexpected behavior indicates that the system is either at a lower temperature than 340 K, or possibly there is also some degree of agglomeration of the nanoparticles. Better understanding the origin of the reduced nanoparticle mobility can be revealed by analyzing the spatial distribution of particles by CXDI.

As the SNR of a single diffraction pattern is too low to reconstruct by CXDI, data were summed over multiple diffraction patterns using a sliding temporal window (see SI) in order to enable reconstruction convergence. Reconstructable data sets for both temperatures were initially generated by summing together 50 diffraction patterns, corresponding to a temporal resolution of 70 ms. These data sets were then reconstructed using PyNX,^26^ where further details can be found in the SI. For the data at 340 K, a second data set was then generated by summing together 16 diffraction patterns, corresponding to a temporal resolution of 22 ms. In the reconstruction of the second data set, the final support functions from the first data set were used as an initial guess. Representative reconstructed CXDI amplitude images, cropped to a size of 4 × 4 μm^2^ around the center and extracted over the same temporal range as the results shown in Figure 1, are shown in Figure 2a,b for the temperatures of 293 and 340 K, respectively. Further details on the data preprocessing and reconstruction steps, as well as reconstructed movies of the data sets, can be found in the SI.

**Figure 2:**
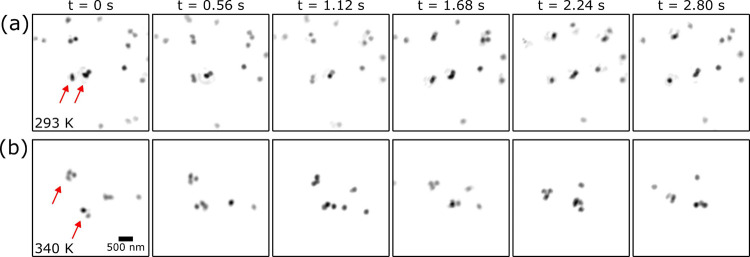
Representative reconstructed CXDI amplitude images at 293 K (a) and 340 K (b), over the same temporal range as the XPCS analysis shown in Figure 1. Images were cropped to a size of 4 × 4 μm^2^ around the center. Before reconstruction, data were summed over 50 frames (293 K) and 16 frames (340 K) which correspond to temporal resolutions of 70 and 22 ms, respectively. The red arrows point to the presence of agglomerations. The scalebar applies to all CXDI images.

For both temperatures, we can identify the presence of individual nanoparticles as well as agglomerates (indicated by the red arrows in Figure 2), all of which undergo Brownian motion. For the 293 K case, there exists two agglomerations consisting of two particles, with the rest of the particles being individual and no particle–particle interactions occurring. In contrast, at 340 K, we not only see faster movement from the particles and the presence of multiple agglomerations, but additionally we can see the particles aggregating over time (see SI movie 2). Near the beginning of the measurement there exists two three-particle agglomerates, but after ≈20 s (see SI movie 2) there appears to be two large agglomerations consisting of roughly seven to eight nanoparticles each. The existence of these agglomerations directly leads to a reduction in the diffusion coefficient, which contributes to the explanation of the discrepancy between the calculated and observed diffusion coefficients obtained by XPCS at 340 K.

For PEGylated Au nanoparticles in glycerol–water mixtures, it has been observed that glycerol can outcompete PEG for the available water molecules in the system, leading to attractive PEG–PEG interactions, which then results in nanoparticle agglomeration.^27,28^ This attraction between the PEG ligands can explain the existence of agglomerations at both temperatures observed here. Further study is required to understand in more detail the agglomeration process observed at 340 K, and in particular, whether the elevated temperature or radiation damage effects cause the process to be enhanced.

The correlations obtained by the XPCS analysis for the full 340 K data set (see SI) shows no clear indication of the onset of the agglomeration process. This highlights the complementary nature of combining both CXDI and XPCS simultaneously. These results have implications for the use of nanoparticles as contrast agents in biological environments,^29,30^ and particularly for examining induced agglomeration processes.^9^

The dynamics identified in the CXDI reconstructions can be better understood by performing single particle tracking analysis, where particle tracking algorithms can identify the particle locations for each point in the time-series.^31^ The mean-squared displacement (MSD), ⟨Δ*r*^2^ ⟩, of the individual particles can be related to the diffusion coefficient by^31^ ⟨Δ*r*^2^⟩ = 2*nDτ*, where *n* is the dimensionality, providing a comparison to those obtained by XPCS. As the CXDI images are two-dimensional projections, *n* = 2, and the MSD becomes ⟨Δ*r*^2^⟩ = 4*Dτ*. The degree of uncertainty was estimated using the standard error^32^ which is dependent on the number of tracked particles.

Figure 3 shows the single particle tracking results extracted over the same time interval as shown in Figure 2, where details on the parameters and error estimation are found in the SI. The obtained estimates of the diffusion coefficients are *D*_293_^CXDI^ = 4,773 ± 1,223 nm^2^/s, and *D*_340_^CXDI^ = 33,222 ± 15,053 nm^2^/s. These values, within error bars, coincide with those obtained by XPCS, but are slightly overestimated. Overestimation of the values can be partially attributed to a number of factors such as misalignment between frames, missing particles in the reconstruction, or blurred motion leading to misidentified particle positions.

**Figure 3:**
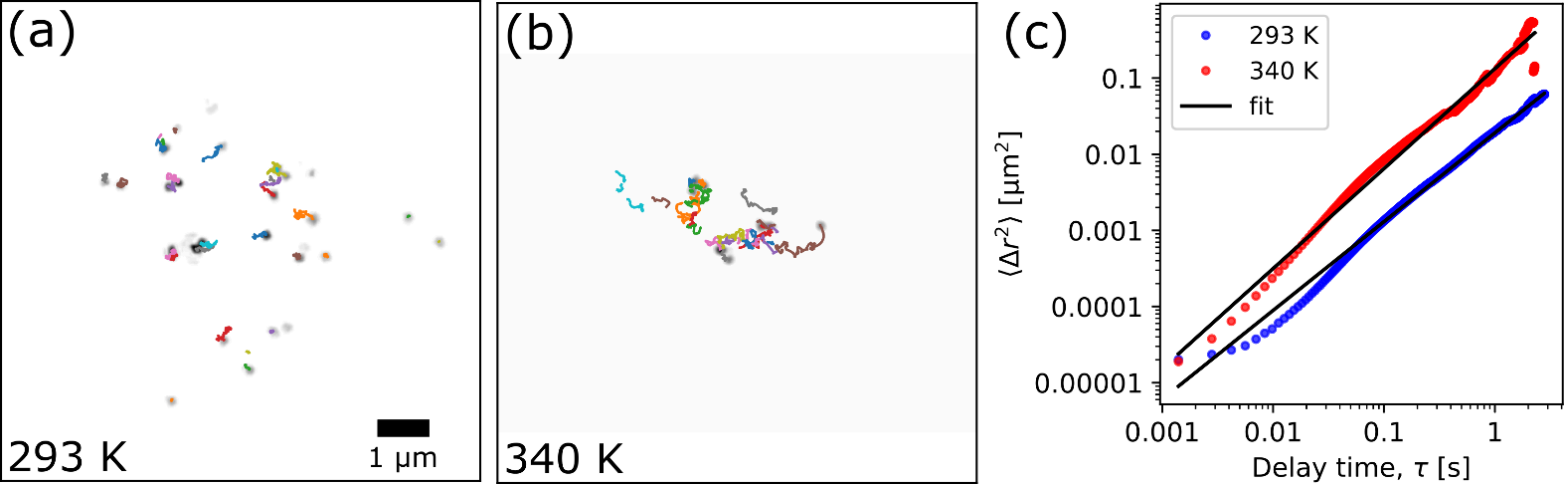
Single particle tracking results from the CXDI images shown in Figure 2 present the motion of the individual particles over 3 s at the beginning of the data set at temperatures of 293 K (a) and 340 K (b). (c) The gradient of the ensemble mean-squared displacement provides estimates of the diffusion coefficient of *D*_293_ = 4,773 ± 1,223 nm^2^/s, and *D*_340_ = 33,222 ± 15,053 nm^2^/s. The uncertainty in the single particle tracking results was estimated using the standard error. The scalebar in (a) applies to both images.

The main consideration in the accuracy of the single particle tracking analysis revolves around the relationship between the quality of the reconstructions, the temporal resolution and SNR, as well as the degree of dynamics of the sample. To investigate the relationship further between these variables and more generally the limits on the ability to perform dynamic CXDI experiments, we analyzed the reconstruction quality as a function of SNR. This was achieved by first generating multiple data sets each of which consist of the integration over a varying number of diffraction patterns using a sliding temporal window. Next, the SNR was calculated for each data set using eq (S5) in the SI, and then reconstructions of each data set were performed. The results are shown in Figure 4, where further details of the SNR calculations and reconstruction process are found in the SI.

**Figure 4:**
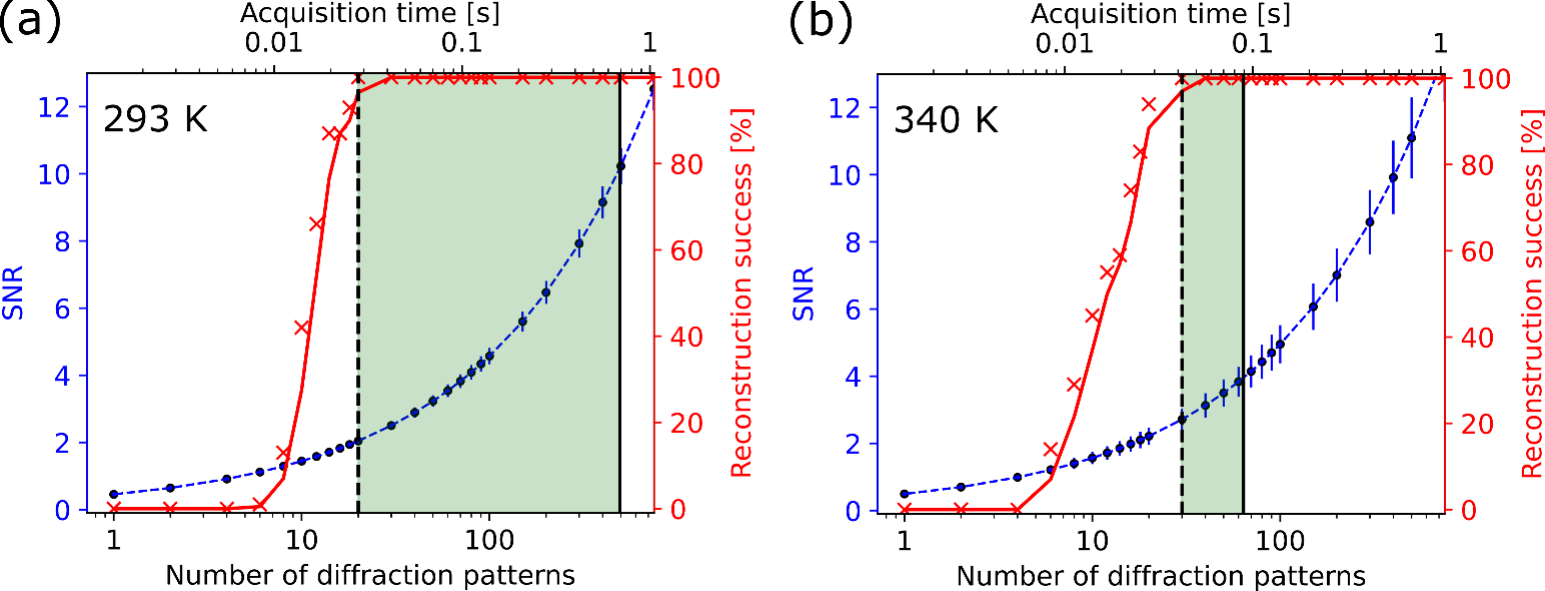
Signal-to-noise ratio (SNR) analysis for reconstructing time-series CXDI data sets at 293 K (a) and 340 K (b). The left axis in blue corresponds to the SNR value, while the right axis in red corresponds to the percentage of times a reconstruction was successful using PyNX. The black dashed line represents the lower limit to the accessible time range determined by the point at which the reconstruction success is 100%. The corresponding SNR values at this limit are SNR = 2.0 (293 K), and SNR = 2.7 (340 K). The critical sampling frequency, *f*_*c*_, is marked by a solid black line. The green shaded region between these lines represents the temporal space accessible by CXDI.

The blue curve in Figure 4 represents the SNR values which increase with an increasing number of summed frames while the red curve represents the percentage chance that a reconstruction will converge. The solid vertical black lines represent the critical sampling frequency, *f*_*c*_, which are the points at which the integration of diffraction patterns is too high such that the object dynamics may become blurred. These were estimated to be *f*_*c*_ = 1.45 Hz (≈493 diffraction patterns) at 293 K, and *f*_*c*_ = 11.21 Hz (≈64 diffraction patterns) at 340 K. These values represent an upper limit to the integration and are dependent on the sample dynamics (see the SI for how these values were estimated). At low numbers of summed diffraction patterns, there is not enough signal to allow the iterative algorithms to consistently converge to a reasonable solution. As the number of summed diffraction patterns increases, there becomes a SNR value which results in a converged reconstruction 100% of the time. This point is taken as a lower limit for reconstructing dynamic CXDI data, and is indicated in Figure 4 as a vertical black dashed line. We see that this limit is reached at a SNR around 2.0 to 2.7, corresponding here to temporal resolutions of 28 and 42 ms, respectively. While it is possible to obtain a reconstruction integrating a smaller number of diffraction patterns, an increasingly large amount of time is required to obtain a good reconstruction. For very large data sets, which will soon become routine, this becomes unreasonable using standard approaches. Future efforts into developing more robust CXDI reconstruction approaches with low SNR is required to push the ability to reconstruct data below this limit.

This work opens up the possibility for many future experimental studies, including exploring structural changes of catalysts *in situ* and *operando*,^15,33^ studying the complex dynamics of nanoparticle self-assembly *in situ*,^34^ as well as distinguishing chirality in self-assembled nanostructures.^35^

To summarize, we have demonstrated simultaneous CXDI and XPCS to study the Brownian motion of colloidal gold nanoparticles with a temporal resolution of 22 ms. The simultaneous analysis allows complementary information to be extracted, where we observed Brownian motion at temperatures of 293 and 340 K, as well as the process of agglomeration at a temperature of 340 K.

As the scattering power of the sample directly relates to the ability to reconstruct a diffraction pattern, we investigated the effect of SNR on the reconstruction performance to provide an estimate of the spatiotemporal space accessible for CXDI. We determine that a lower limit is reached at SNR ≈ 2. This can act as a guide for the design of future *in situ* and *operando* experiments to study a wide range of dynamic systems.

The results demonstrated here were enabled by recent advancements in hybrid photon counting detectors technology, namely; fast frame rate, double-buffering to readout frames with zero deadtime, high dynamic range from single photon counting to over 1e6 photons/pixel/sec. To image dynamic systems using coherent diffractive techniques on faster time scales will require future improvements in detector technology, to take advantage of the increased brightness at diffraction limited light sources, or further development of novel reconstruction approaches (e.g., ref (20)).

## Supporting Information Available

Additional experimental details, and detailed data processing steps for XPCS, CXDI, and single particle tracking analyzes (PDF)
Reconstructed amplitude movie for 293 K data (MP4)
Reconstructed amplitude movie for 340 K data (MP4)
