# Supplementary material for: Dynamic X-ray Coherent Diffraction Analysis: Bridging the Time Scales between Imaging and Photon Correlation Spectroscopy
Source: Nano Lett. 2024 Oct 18;24(43):13702–7. doi: 10.1021/acs.nanolett.4c03699 (PMC11528431; doi:10.1021/acs.nanolett.4c03699)
Supplement: Supplementary file 1 — nl4c03699_si_001.pdf [file nl4c03699_si_001.pdf]

Supporting Information

Dynamic X-ray Coherent Diffraction Analysis: Bridging the Time Scales between Imaging and Photon Correlation Spectroscopy

Gerard N. Hinsley,[*,†] Fabian Westermeier,[†] Bihan Wang,[†,‡] Kuan Hoon Ngoi,[†] Shweta Singh,[†] Rustam Rysov,[†,⊥] Michael Sprung,[†] Cameron M. Kewish,[¶,§] Grant A. van Riessen,[§,‖] and Ivan A. Vartanyants[*,†]

†*Deutsches Elektronen-Synchrotron DESY, Notkestr. 85, Hamburg 22607, Germany*

‡*Center for Transformative Science, Shanghai Technical University, Shanghai 201210, China*

¶*Australian Nuclear Science and Technology Organisation, Australian Synchrotron, Victoria 3168, Australia*

§*Department of Mathematical and Physical Sciences, La Trobe University, Bundoora, Victoria 3086, Australia*

‖*Melbourne Centre for Nanofabrication, Clayton, Victoria 3168, Australia*

⊥*Present address: European X-ray Free Electron Laser Facility, Holzkoppel 4, Schenefeld 22869, Germany.*

E-mail: gerard.hinsley@desy.de; ivan.vartaniants@desy.de

Sample description

Au nanoparticles were obtained from Nanopartz (Loveland, US).[1] The nanoparticles have a diameter of 200 nm, are functionalised with 3 kDa poly(ethylene glycol) methyl, and were dispersed in a solution of 18 MEG DI water. This acts as the stock solution, where the weight concentration of nanoparticles is 3.9 mg/mL, and the volume fraction is approximately 0.02%.

A volume of 100 µL of the stock solution was extracted and centrifuged at 5000 rpm for 5 minutes. The liquid was then aspirated, and the same volume of glycerol was added. This was then sonicated to redisperse the particles. From this, 10 µL of the solution was extracted and combined with 90 µL of glycerol, diluting the solution, before the sample was re-sonicated to again disperse the nanoparticles uniformly within the solution.

Rectangular capillaries with dimensions 0.5 mm $\times$ 5 mm $\times$ 0.05 mm (H $\times$ V $\times$ W) were then filled with the solution, and sealed to enable vacuum compatibility.

From Ref. 2, we can expect that the length of the ligand is between 8 – 14 nm in water. This length will change due to many factors, such as the presence of glycerol, changes in temperature, and the grafting density. Understanding the influence of each of these parameters on the aspects of the ligand properties is out of the scope of the current manuscript.

Experiment and data pre-processing

The experiment was performed at the P10 Coherence Applications beamline at PETRA III, as shown in Fig S1. The coherent source size of the incident 8 keV X-rays was controlled by apertures which were set to a size of 80 $\times$ 125 µm^2 (H $\times$ V). This was then focused using compound refractive lenses to a size of 2.8 $\times$ 2.3 µm^2 (H $\times$ V) at full width half maximum (FWHM). The capillary containing the nanoparticles was placed at the focal plane, and the detector was placed 5 m downstream of the sample. Diffraction patterns were recorded at 714 Hz using an EIGER 4M detector, which has pixels of size 75 $\times$ 75 µm^2. A

tungsten cylinder was used as a beamstop to block the central beam, and was attached to a semi-transparent beamstop consisting of two Si wafers of dimensions 3 mm × 3 mm × 100 µm, and 5 mm × 5 mm × 100 µm (H × V × W).

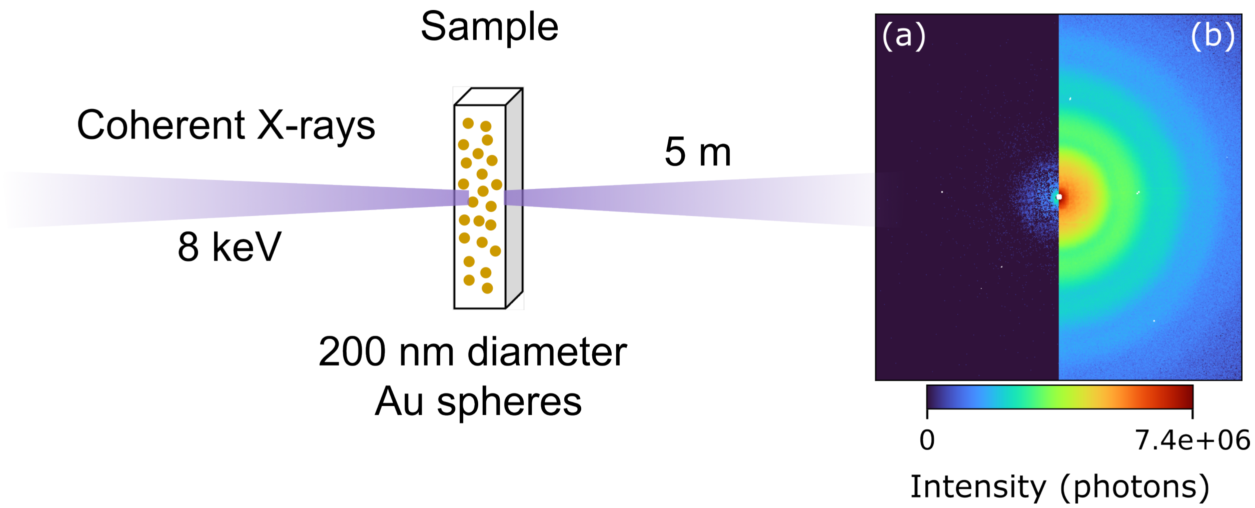

Figure S1: A diagram of the experimental setup, where coherent X-rays with an energy of 8 keV are focused to a size of 2.8 × 2.3 µm^2 (H × V) at the focal plane. The capillary containing the dispersion of the 200 nm diameter Au nanoparticles was positioned at the focal plane. Diffraction patterns were measured 5 m downstream of the sample position. The diffraction pattern shows one acquisition (a) and the summation of all diffraction patterns (b).

Data were prepared for reconstruction by cropping diffraction patterns to a size of 480 pixels × 480 pixels around the beam center. The intensities of the pixels affected by the semi-transparent beamstop were then scaled by multiplying the values by the expected X-ray absorption at 8 keV. A representative image of a single diffraction pattern after pre-processing is shown in Fig. S2(a), while (b) and (c) show diffraction patterns after summing together 16 and 50 frames, respectively. A pixel mask was simultaneously created to remove dead pixels, and the effect of the tungsten beamstop for which a radius of 4 pixels at the beam center were excluded. To reduce the influence of the probe profile in the reconstruction, a larger central square region was included in the mask. The final mask used for reconstruction is shown in Fig. S2(d).

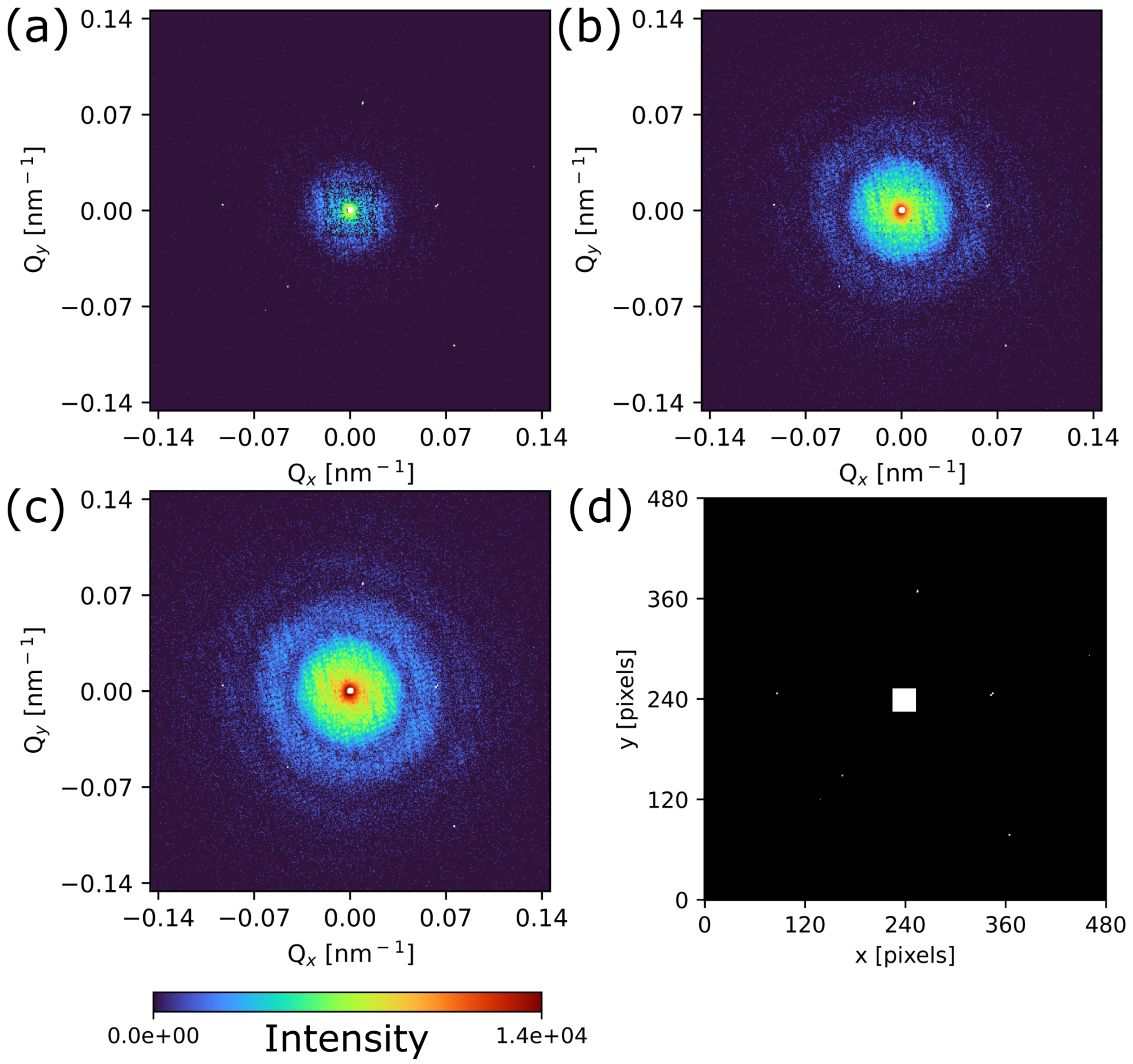

Figure S2: (a)-(c) Images of the recorded diffraction patterns after post-processing. (a) a single diffraction pattern from the 293 K data. (b) Diffraction pattern after summing together 16 frames. (c) Diffraction pattern after summing together 50 frames. (d) The pixel mask used in the reconstructions. White pixels in (a)-(c) represent bad pixels with incorrect intensities, while (d) includes extra pixels which were added to additionally mask intensities related to the probe. Masked pixels were allowed to fluctuate freely in the reconstruction.

Temporal window

For a time series data set we collect $n = 1...N$ diffraction patterns $I(q,t)$, where in this experiment $N = 21,000$. To generate a reconstructable data set, $I^R(q,t)$, from this time-series, we sum together frames using a sliding temporal window of width x. Within $I^R(q,t)$, the intensity of the nth diffraction pattern is then given by

$$I_n^R(q,t) = \sum_{i=n-x/2}^{n+x/2} I_i(q,t). \tag{S1}$$

When performing the reconstructions, we excluded frames where $n < x$ and $N - n < x$, ensuring that each reconstructed frame was treated equally. For the results shown in Fig. 2 in the main text, the final $I^R(q,t)$ data sets were generated using temporal window widths of $x = 50$ and $x = 16$, for the temperatures of 293 K and 340 K, respectively.

XPCS data analysis

The second-order autocorrelation $g^{(2)}(q,\tau)$ function is calculated by

$$g^{(2)}(q,\tau) = \frac{\langle I(q,t)I(q,t+\tau)\rangle}{\langle I(q,t)\rangle^2}, \tag{S2}$$

where $I(q,t)$ is the measured intensity, q is the modulus of the wave-vector, t is time, and τ is the delay time. The results from calculating $g^{(2)}(q,\tau)$ over the whole time-series is shown in Fig. S3(a,b), for temperatures of 293 K and 340 K, respectively. We see that the $g^{(2)}(q,\tau)$ correlation functions shown in Fig. S3 exhibit two decays representing fast and slow dynamics. The fast dynamics are attributed to Brownian motion, which can be fitted using

$$g^{(2)}(q,\tau) = \alpha + \beta \exp\left[-2(\Gamma\tau)^\gamma\right], \tag{S3}$$

where α is the baseline, β is the speckle contrast, Γ is the relaxation rate, and the exponent γ is a measure of the distribution of relaxation times.[3] The final values of α and β obtained

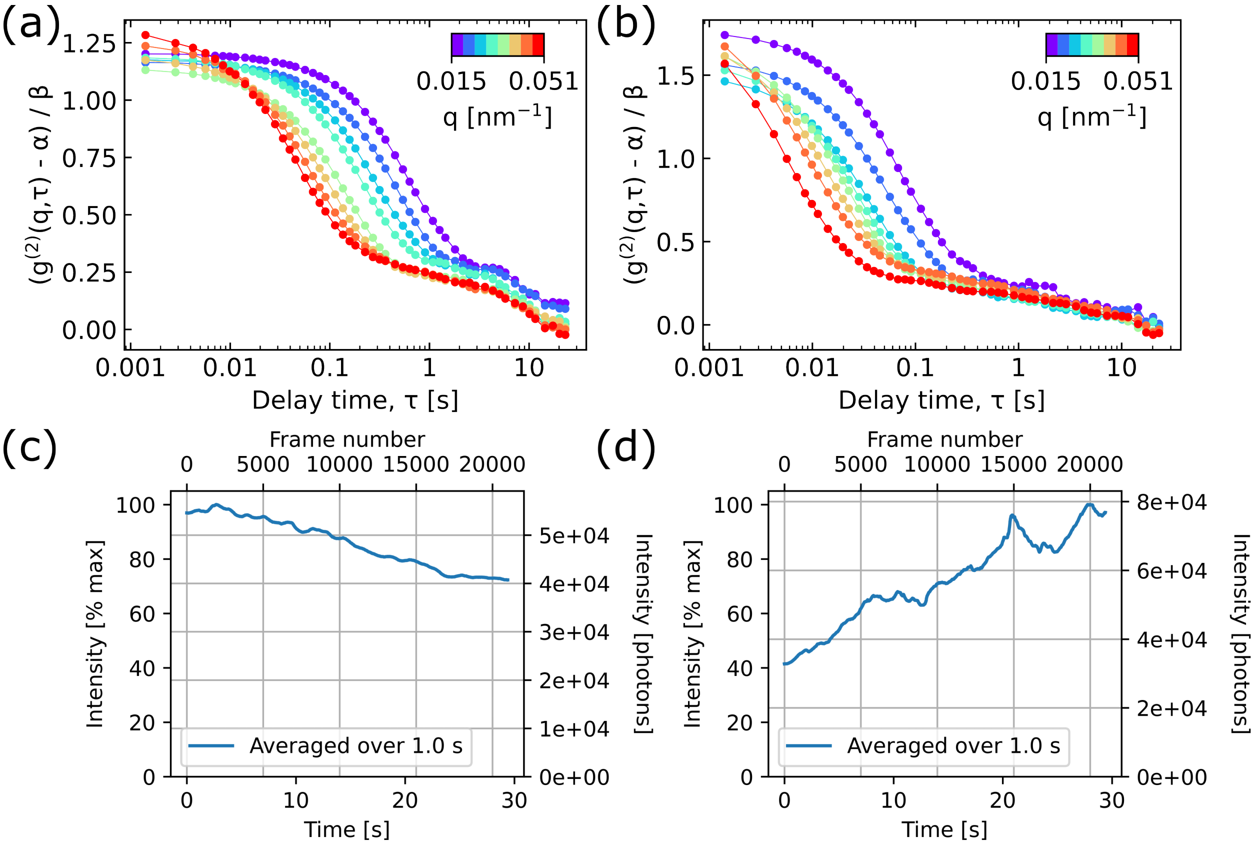

Figure S3: Experimental $g^{(2)}(q, \tau)$ functions as a function of delay time at temperatures of 293 K (a) and 340 K (b) over the whole time series. The color represents the eight different q-partitions used for the analysis, where the partitions are spaced with equal dq/q steps. The α and β used for normalization were obtained from the fits of the data shown in Fig. 1 in the main text. The total scattered intensity smoothed over 1 s as a function of time for 293 K (c) and 340 K (d). The change in intensity directly relates to the number of particles within the field-of-view.

from the fitting can be seen in Fig. S4, while the values of Γ are found in Fig. 1 in the main text, and $\gamma = 1$ was used for the fits of both temperatures as we expect the motion to be Brownian.

Results of fits when letting γ be a free parameter are shown in Fig. S5. We see that the fits at 293 K are all reasonable, with $\gamma \approx 1$ for all q-partitions. On the other hand, the fits of the data at 340 K are poor where γ constantly decreases at larger q, which is not expected for a system undergoing Brownian diffusion. Due to this, we used the result of $\gamma = 1$ at 293 K for the fitting analysis at both temperatures to ensure the analysis of both data sets was identical.

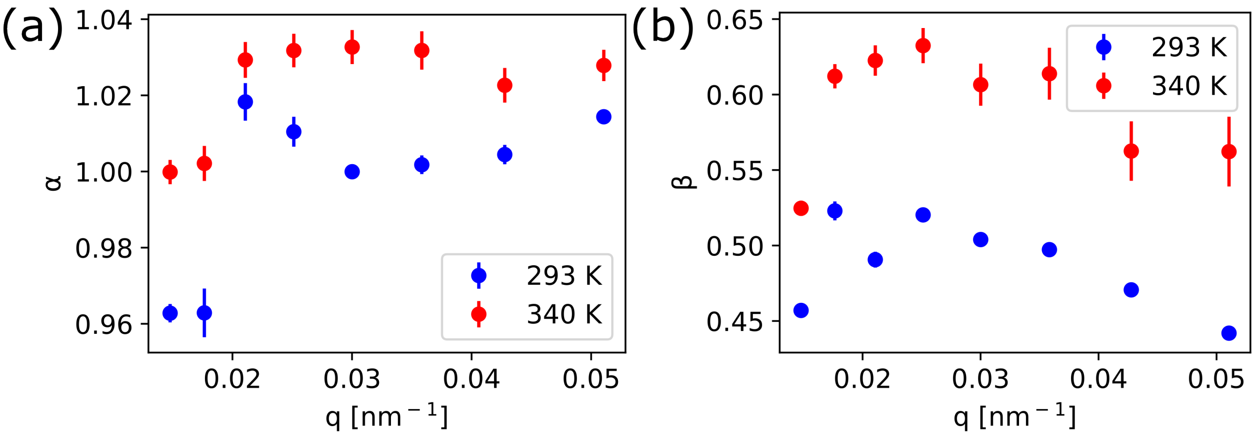

Figure S4: The values of (a) the baseline, α, and (b) the speckle contrast, β, obtained by fitting the $g^{(2)}(q, \tau)$ functions shown in Fig. 1 in the main text.

The decay representing slow dynamics we attribute to changes in the scattered intensity, which can be seen in Fig. S3(c,d) for 293 K and 340 K data, respectively. Due to this, we limited the fitting analysis to a smaller time range in which the intensity changes remained relatively constant.

The fluctuating intensity is a source of error in the XPCS results and stems from the limitations of the experiment when trying to simultaneously combine CXDI and XPCS. In order to simultaneously employ CXDI, the beamsize was reduced to oversample the speckle, and the number of particles was reduced to allow tracking of individual particles, compared to typical XPCS experiments. A consequence of this is that a change of a single

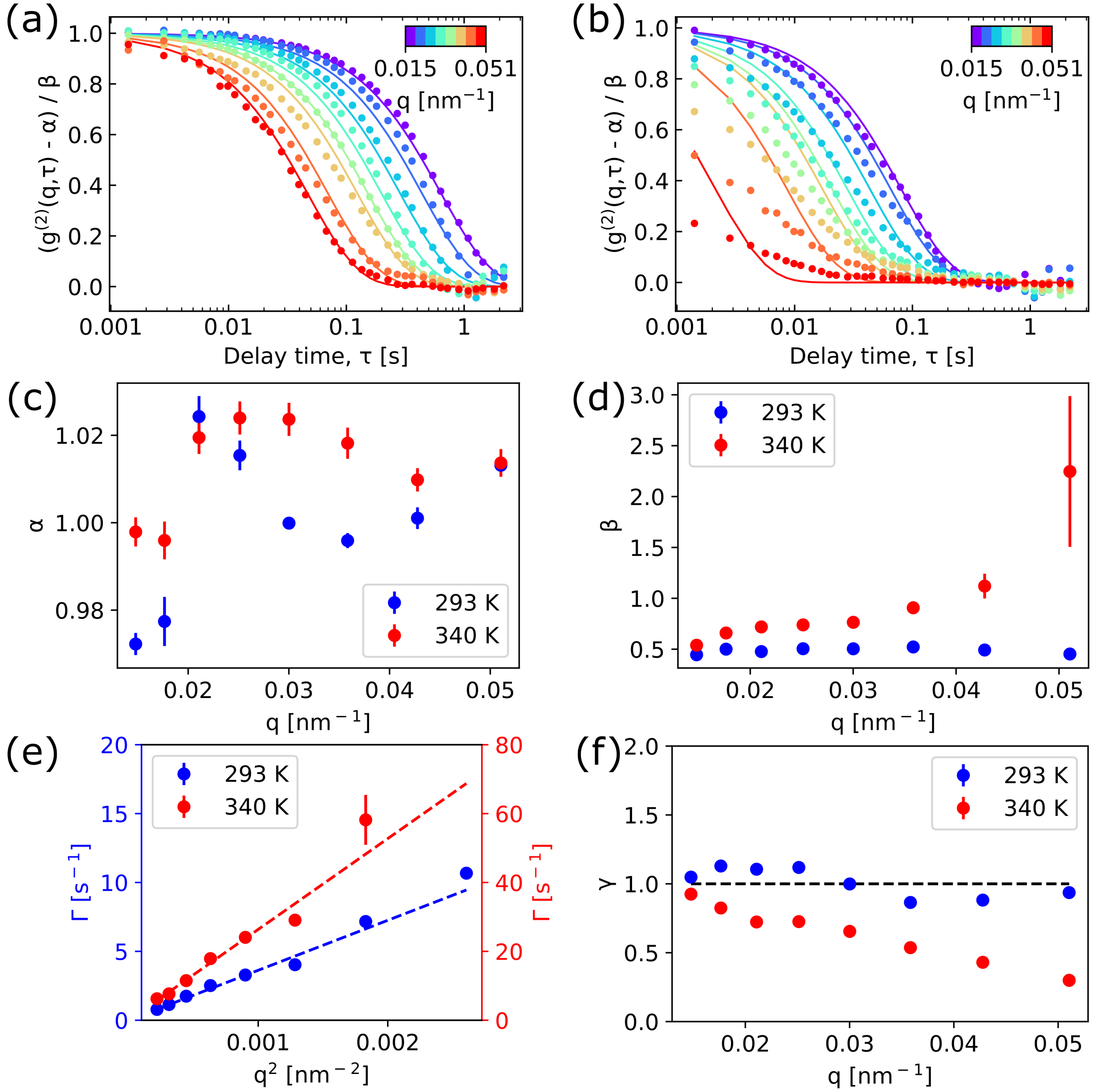

Figure S5: Fitting results of both data sets when γ is set to be a free parameter. (a,b) Experimental $g^{(2)}(q,\tau)$ functions (points) and their fits (solid lines) as a function of delay time at temperatures of 293 K (a) and 340 K (b) over 3 s of data near the beginning of the series. The results of the baseline α and the contrast β in the displayed fits were used for the normalization of the $g^{(2)}(q,\tau)$ functions. The color represents the eight different q-partitions used for the analysis, where the partitions are spaced with equal dq/q steps. The values of (c) the baseline, α, (d) the speckle contrast, β, (e) the relaxation rate, Γ, and (f) γ obtained from the fitting.

nanoparticle in the field-of-view (FOV) can lead to a significant change in the scattered intensity. As XPCS typically employs a larger beamsize and has a larger concentration of particles, the $g^{(2)}(q,\tau)$ correlation curves are insensitive to a few particles entering or leaving the FOV. This experiment demonstrates that although CXDI has a more stringent experimental configuration than XPCS which reduces the signal-to-noise ratio (SNR), there is still overlap between an optimal imaging regime for CXDI, and suitable parameters for XPCS.

The $g^{(2)}(q,\tau)$ analysis above calculates the mean correlation between all diffraction patterns of the time series for a given τ. To calculate the correlation between multiple time points, we can use the two-time correlation function, $C(q,t_1,t_2)$, which is calculated as

$$C(q,t_1,t_2) = \frac{\langle I(q,t_1)I(q,t_2)\rangle}{\langle I(q,t_1)\rangle\langle I(q,t_2)\rangle}. \tag{S4}$$

The results from the two-time correlation analysis are shown in Fig. S6(a-c) and (d-f), for temperatures of 293 K and 340 K, respectively. Three different q-partitions are shown, corresponding to values of (a,d) 0.018 nm^{-1}, (b,e) 0.021 nm^{-1}, and (c,f) 0.025 nm^{-1}. Although we see the process of agglomeration in the CXDI reconstructions, the two-time correlations appear to be relatively constant with only minor changes to the correlations over time. In each two-time correlation map, there appears to be fluctuations in the low correlated regions. As these fluctuations are not consistent with different q-partitions, we attribute these fluctuations to being an artefact due to the low number density of particles. They do not represent a physical process occurring within the sample. This is confirmed by simulations shown in Fig. S11, where similar fluctuations can be observed.

CXDI reconstruction process

The PyNX package[4] was used to perform the iterative algorithm process which consisted of a sequence of 2000 relaxed averaged alternating reflections (RAAR)[5] + 500 Error Reduction

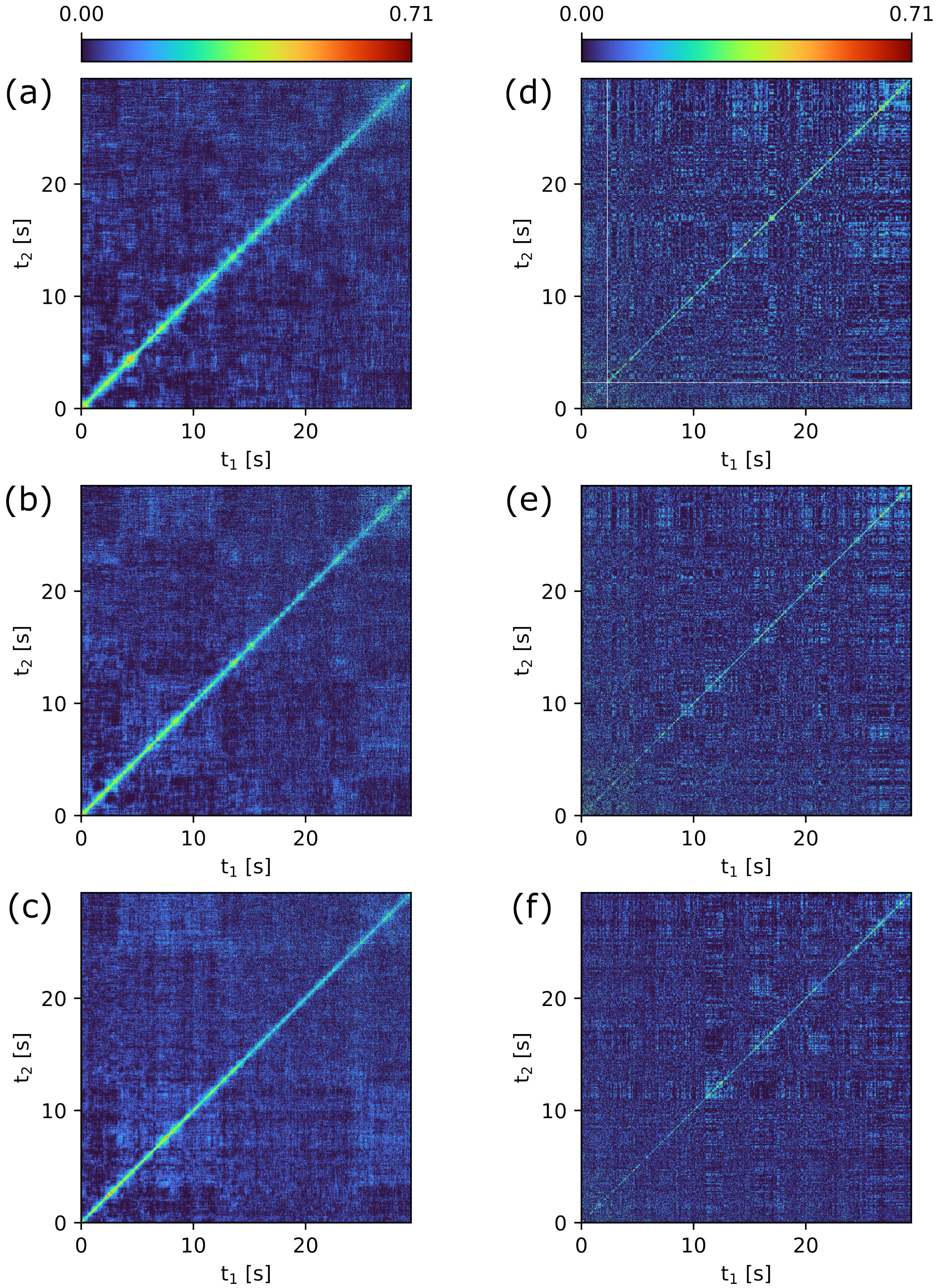

Figure S6: Plots of the two-time correlation maps calculated for 293 K (a-c) and 340 K (d-f). The values of q at which correlations were calculated are 0.018 nm^{-1} (a,d), 0.021 nm^{-1} (b,e), and 0.025 nm^{-1} (c,f). All maps are shown on the same color scale.

(ER) iterations.[6] Modulus and support constraints were applied every iteration, with the shrinkwrap algorithm[7] applied every 150 iterations. Pixels within the mask were allowed to fluctuate and were not constrained. Partial coherence correction through the Lucy-Richardson deconvolution,[8] as implemented in PyNX, was used.

Reconstructions were performed serially. The first time point was reconstructed using the autocorrelation as the starting support. For subsequent time points, the final support from the previous time point was used as the starting support.

To begin, data at both temperatures were generated by using a temporal window with length $x = 50$ diffraction patterns, and were subsequently reconstructed as described above. To reconstruct the data at 340 K at a SNR below the 'limit' indicated in the main text, providing reconstructed images with less blurred motion, a new data set was generated with a temporal window length of $x = 16$ diffraction patterns. The final supports from the reconstruction of the 50 diffraction pattern data was then used as a starting support for the 16 diffraction pattern data and the supports were then held constant throughout the iterative process.

After all reconstructions were performed, any twin images within the data set were manually inverted such that the entire data set reconstructed the same upright object. Images were then aligned using the StackReg[9] plugin within ImageJ.

With a volume fraction of 0.02% in the stock solution, and a 1:10 dilution, we expect that the percentage of the reconstructed volume containing a particle is 0.002%. If we assume a reconstructed volume of $5 \times 5 \times 50 = 1250$ µm^3, the volume containing particles is then 2.5 µm^3. One nanoparticle has a volume of approximately 4.2×10^{-3} µm^3, so the number of particles we expect to see is approximately 600. This is not reflected in our data, as we observe at most around 20 nanoparticles at once. We attribute this difference to agglomeration occurring with the stored nanoparticles. Agglomeration during storage of the nanoparticles then leads to some agglomerations to sediment and fall out of the solution, resulting in a lower effective concentration of the stock solution. This also helps to explain

the observation of agglomerates at 293 K.

Signal-to-Noise Analysis

Data sets with different SNR values were generated by using temporal windows of different sizes, the smallest being $x = 1$ and the largest being $x = 750$. For a given value of temporal window length x, the SNR is calculated as:

$$\mathrm{SNR} = \left\langle \frac{I^R(q,t)}{\sqrt{I^R(q,t)}} \right\rangle_n , \tag{S5}$$

where $\langle \cdots \rangle_n$ denotes averaging over all diffraction patterns. The uncertainty of the SNR was estimated by the standard deviation. The SNR was calculated while only ignoring bad pixels and those corresponding to the beamstop within the diffraction pattern, *i.e.*, we did not exclude the central pixels related to the probe in the calculation.

As poor SNR prevented the successful reconstruction of some frames, reconstructions for a given data set were attempted using PyNX 5 times. By default PyNX uses five tries to reconstruct a data set per attempt, leading to a possible 25 trials for a given data set. The criteria for success was taken to be convergence of the CDI reconstruction per attempt, not for each trial.

Computational time of the reconstructions was shortened by only reconstructing every 1,000th frame instead of all 21,000 frames, producing 20 unique data sets for a given SNR value. The final values of success percentage are then the average of all 20 data sets.

Particle tracking

Particle tracking was performed in Python using the TrackPy[10] package. The following TrackPy parameters were used to identify particles within each individual frame: diameter = 11 pixels, the minimum integrated brightness = 1000, maximum radius-of-gyration of

brightness = 10.0, and the minimum separation between features = 4 pixels. For linking trajectories, the following parameters were used: max displacement = 5 pixels per frame, memory = 21000 frames. Trajectories which were identified for less than 10 frames were ignored. Drift within the time-series was eliminated using the built-in function within TrackPy before calculating the mean-squared displacement, $\langle \Delta r^2 \rangle$. This was then related to the Diffusion coefficient, D, by

$$\langle \Delta r^2 \rangle = 4D\tau. \tag{S6}$$

The uncertainty in the single particle tracking results was estimated by the standard error[11]

$$\sigma = \frac{1}{\sqrt{P}}, \tag{S7}$$

where σ is the uncertainty, and P is the number of tracked particles. The number of frames for which a particle may be tracked will depend on when a particle enters or leaves the field of view, if there is any overlap with another particle, or also the presence of artefacts in the reconstruction obscuring the ability to identify a particle. Due to this, the number of tracked particles P decreases for large number of tracked frames, leading to an increase in the uncertainty. The number of tracked particles, and therefore an estimate of the uncertainty, as a function of tracked frames is shown in Fig S7(a,b) for the data at 293 K and 340 K, respectively.

To provide a single estimate of the uncertainty on the calculated diffusion coefficients using single particle tracking, we use the mean value of σ across all frames. This leads to uncertainties of σ_{293} = 25.6% ($\pm 1,223$ nm^2/s), and σ_{340} = 45.3% ($\pm 15,053$ nm^2/s). We note that the uncertainty in the results at 340 K are quite high. As the number of particles tracked for more than 1000 frames for the 340 K case is quite small, it may be more accurate to estimate the uncertainty over only 1000 tracked frames. Doing this results in σ_{340} = 28.9%

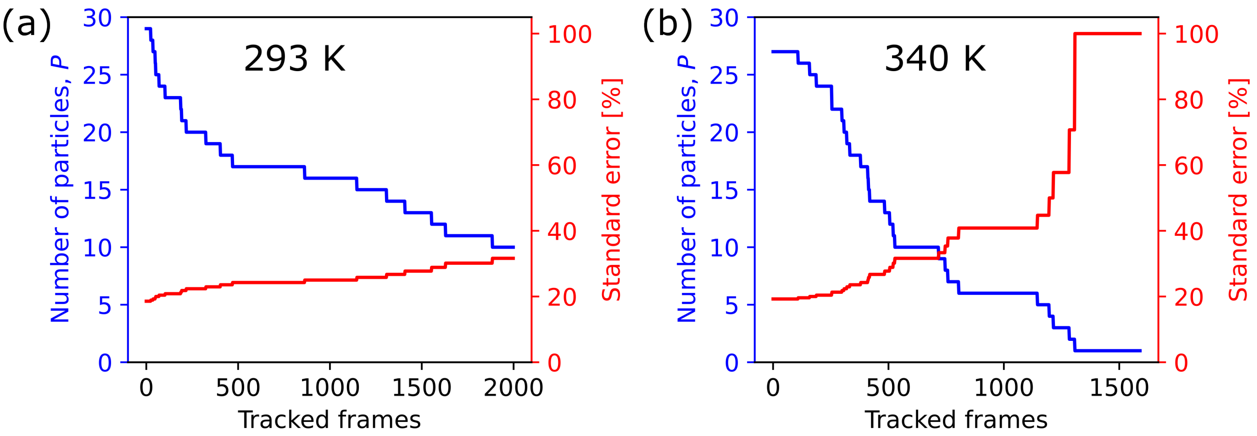

Figure S7: Single particle tracking uncertainty using the standard error for data at 293 K (a) and 340 K (b). The blue lines represent the number of particles identified, while the red line is the corresponding standard error.

($\pm 9,603$ nm^2/s), where the agreement between D_{340}^{CXDI} and D_{340}^{XPCS} is still within error.

Critical sampling frequency

The critical sampling frequency, f_c, refers to the degree of sampling required in order to fully capture the dynamic behavior of interest. The value of f_c is sample-dependent, and is the inverse of the critical acquisition time, $f_c = 1/\tau_c$. As described in Ref. 12, to estimate a value of f_c for Brownian motion, we can define that the critical acquisition time τ_c is equal to the time required for the mean displacement of a particle to be half it's diameter, $\Delta r = 0.5d$. Substituting this into Eq. (S6) we get:

$$\Delta r^2 = 4D\tau \Rightarrow \left(\frac{d}{2}\right)^2 = \frac{4D}{f_c},$$

and rearranging we get

$$f_c = \frac{16D}{d^2}. \tag{S8}$$

For the 293 K data, $D = 3,618$ nm^2/s, and $f_c = 1.45$ Hz, or equivalently a critical acquisition time of $\tau_c = 691$ ms. For the 340 K data, $D = 28,024$ nm^2/s, and $f_c = 11.21$ Hz,

or equivalently a critical acquisition time of $\tau_c = 89$ ms. Here, we are ignoring the thickness of the ligand shell and using $d = 200$ nm. This is due to the many different aspects which can affect the ligand properties, and therefore the resulting thickness and the hydrodynamic radius of the nanoparticle. Obtaining an accurate value of the hydrodynamic radius is out of the scope of the present manuscript. As we expect the ligand to be no larger than 14 nm,[2] which is much smaller than the diameter of the Au core, we can expect that the main source of error in the final value is attributed to the standard error of estimating D. There is also uncertainty in the actual value of the viscosity, which introduces further error into the estimation of the hydrodynamic radius.

Microrheology analysis

The viscosity of a liquid can be calculated by

$$\eta = \frac{k_B T}{3\pi D d}. \tag{S9}$$

Using $D = 3,618$ nm^2/s, the XPCS result at 293 K, the viscosity of the solution is $\eta = 0.59$ Pa s. The composition of the solution is known to be a mixture of water and glycerol, each of which have their own viscosities, η_W and η_G, respectively. Following the process in Ref.,[13] both viscosities may be approximated by:

$$\eta_W = 12100 \exp\left[\frac{T(T-1233)}{9900+70T}\right], \tag{S10}$$

and

$$\eta_G = 1.79 \exp\left[\frac{T(T-1233)}{36100+360T}\right]. \tag{S11}$$

The relative percentage of water can be found through:

$$C_W = \frac{\log(\eta/\eta_G)}{\log(\eta_W/\eta_G)}, \tag{S12}$$

and the relative percentage of glycerol can be found through $C_G = 100\% - C_W$. Using the above equations, we obtain $C_W = 12\%$ and $C_G = 88\%$ at 293 K.

Analysis of $I(q)$

A standard approach to understanding the structural dynamics is by analyzing the $I(q)$. Figure S8 shows the evolution of $I(q)$ over time for both (a) 293 K and (b) 340 K data from 1 s to 28 s. We observe that there are minimal changes in the data at 293 K, and some subtle differences over time at 340 K, which is expected as from the CXDI reconstructed images we are able to observe agglomeration occurring.

To better compare the two temperatures, Fig. S9(a) shows the first and last $I(q)$ at both temperatures from Fig. S8, allowing a direct comparison between time and temperature. The vertical shifts represent changes in the scattered intensity, which correlates with the number of particles within the field-of-view. Excluding this, there are minimal changes in the shape of $I(q)$ for both 293 K curves, as well as the $I(q)$ of 340 K at 1 s. The red curve, corresponding to the 340 K data at 28 s, shows the biggest change where the second and third peaks relating to the spherical form factor appear flatter. This can occur when the polydispersity is increased. There is also some structure factor appearing at a q-value of around 0.023 nm^{-1}, which corresponds to a real space distance of approximately 270 nm. This distance matches what we would expect the distances between particles to be in the agglomerate, as judged from the CXDI reconstructions. We do not, however, see any changes in the overall shape of the form factor. Figure S9(b) shows the expected curve obtained by simulating a single spherical particle form factor where the polydispersity was set to 8%, and the radius of the Au nanoparticle was assumed to be 103 nm. We can see that the position of the peaks of the form factor match closely with that of the azimuthal profile obtained at

293 K for the first second of data.

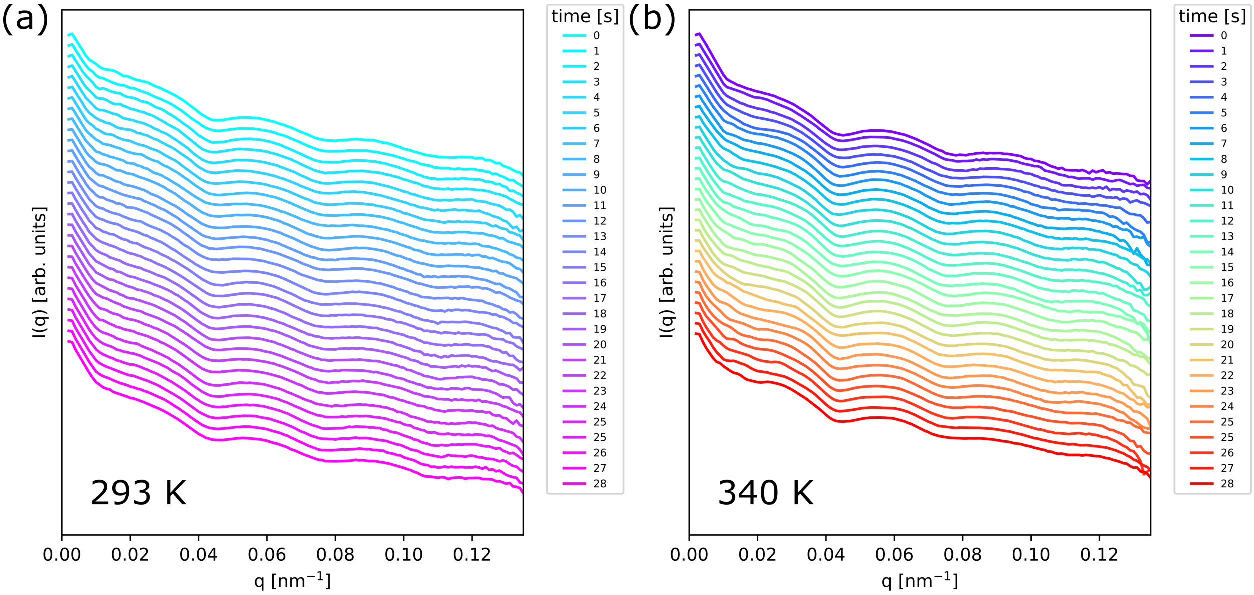

Figure S8: Plots of the evolution of $I(q)$ over time for the data measured at (a) 293 K and (b) 340 K. Azimuthal profiles were obtained by first integrating over 700 frames (approximately 1 s) before obtaining the mean value at each q-partition. The Δq used was 0.001 nm^{-1}. The intensities have been offset vertically for clarity.

Simulation of the experiment

To demonstrate the validity of CXDI to provide accurate information regarding the Brownian motion dynamics, we simulated the experiment presented in this work. We simulated the Brownian motion of Au nanoparticles with a diameter of 200 nm in a 90% glycerol plus 10% water solution at a temperature of 293 K, providing a diffusion coefficient of $D = 3,130$ nm^2/s. We simulated the object arrays with a size of 10×10 μm^2. Within this area, we set the particle concentration to be an area fraction of about 3.6% (122 particles), such that the expected number of particles within the probe FWHM at any given moment in time would be around 5. The starting position of each particle was random, while updated positions were calculated for each time point using a random normal distribution and appropriately scaled by the simulated diffusion coefficient. We generated 21,000 frames, where the time between frames was 1.4 ms. The experiment was simulated at a photon energy of 8 keV, where from

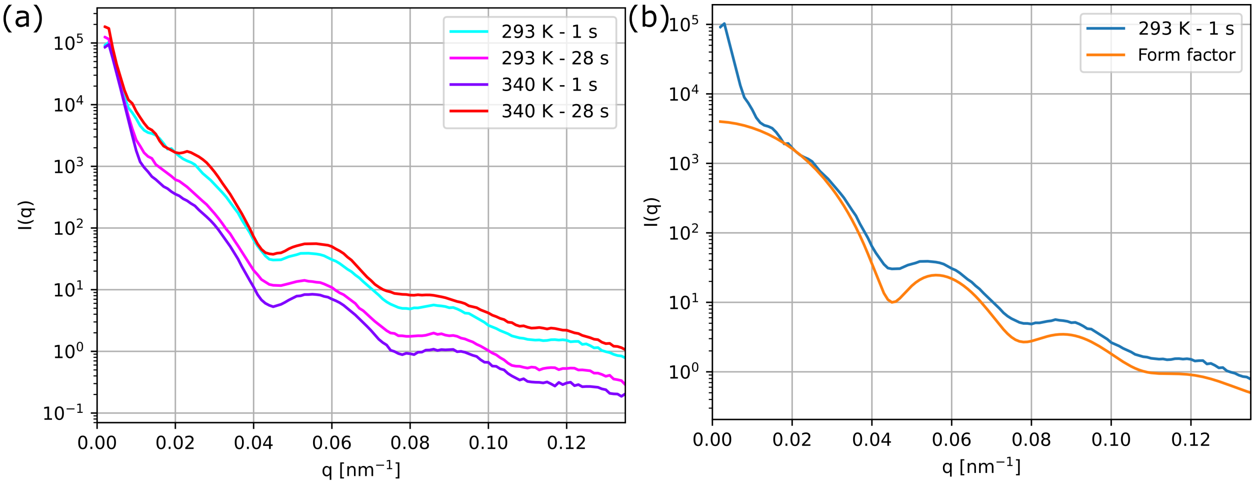

Figure S9: (a) Plots of the first and last azimuthal profile obtained at both 293 K and 340 K. The $I(q)$ are those directly from Fig. S8. (b) Plot of $I(q)$ obtained using the data at 293 K for the first second of data acquisition (blue line) alongside the expected form factor for a single spherical nanoparticle (orange). For the form factor calculation, the polydispersity was set to 8%, and the radius of the Au nanoparticle was assumed to be 103 nm.

Ref. 14, values of the complex refractive index required to generate the object arrays were obtained. We then simulated a Gaussian probe function with a FWHM value of 2.35 µm with uniform flat phase, where the probe was scaled such the flux was 5×10^{10} photons per second – lower than what was obtained during the actual experiment. We obtained the exit-surface wave function through the multiplication of probe and object functions, then Fourier transformed and squared the result to generate diffraction pattern intensities. Noise was then introduced by scaling the measured intensities in individual diffraction patterns by a Poisson distribution. This simulated data set was then analyzed using CXDI and XPCS using similar methodologies as previously described.

The amplitude of the simulated object functions at representative time points is shown in Fig. S10(a), while the corresponding reconstructed amplitudes are shown in (b). Both the simulated and reconstructed images have been cropped to a size of 4×4 µm^2, and for the reconstructed images we have also performed alignment of the time-series. Towards the edges of the reconstructed images, the particle contrast appears to reduce, and at some distance away from the center we can no longer see particles. This behavior of the reconstructions

relates to the position of the particles in reference to the beam position, where the simulated intensity of the probe function is shown in Fig. S10(c). Particles which are within the FWHM of the probe envelope contribute many more scattered photons to the diffraction pattern, while those in the probe tails contribute a relatively small amount. Nevertheless, from the particles which are reconstructed, we can see quite good agreement between the images in regards to the relative particle positions. With sufficient particles reconstructed, we can then employ single particle tracking analysis methods, as shown in Fig. S10(d), which then enable estimation of the mean-squared displacement, shown in Fig. S10(e). The single particle tracking results return an estimated diffusion coefficient of $D = 2,441 \pm 772$ nm^2/s, which is within error of the simulated value of $D = 3,130$ nm^2/s. This result is similar to the result obtained from the experimental data, where the small number of particles tracked introduces an error into the estimated diffusion coefficient. Overcoming these issues in CXDI will depend on clearer reconstructed images which contain more particles. Alternatively, the ability to simultaneously use XPCS allows for a more accurate ensemble estimate of the diffusion coefficient, showing the complementary benefits of this experimental design.

Figure S11 shows results from the simulations examining the effect of the particle number fluctuations on the XPCS analysis. The scattered intensity plotted against time is shown in Fig. S11(a), where we can see that due to the movement of particles within the beam, we have changes of the intensity between 80% and 100% of the maximum. How this fluctuating intensity affects the XPCS results is shown in Fig. S11(b), which shows the calculated $g^{(2)}(q, \tau)$ functions. These functions exhibit multiple features, such as the expected short-time Brownian diffusion, some features corresponding to longer time-scales, and some offset in the baseline. As this system was only simulated with Brownian motion, we can attribute that the longer time-scale correlations relate to the number fluctuations, and therefore do not affect the short time scale analysis. Examining how the fluctuations affect the two-time correlations is shown in Fig. S11(c-f), where there appears to be some intermittencies as was observed in the real data shown in Fig. S6. Again, as we know that

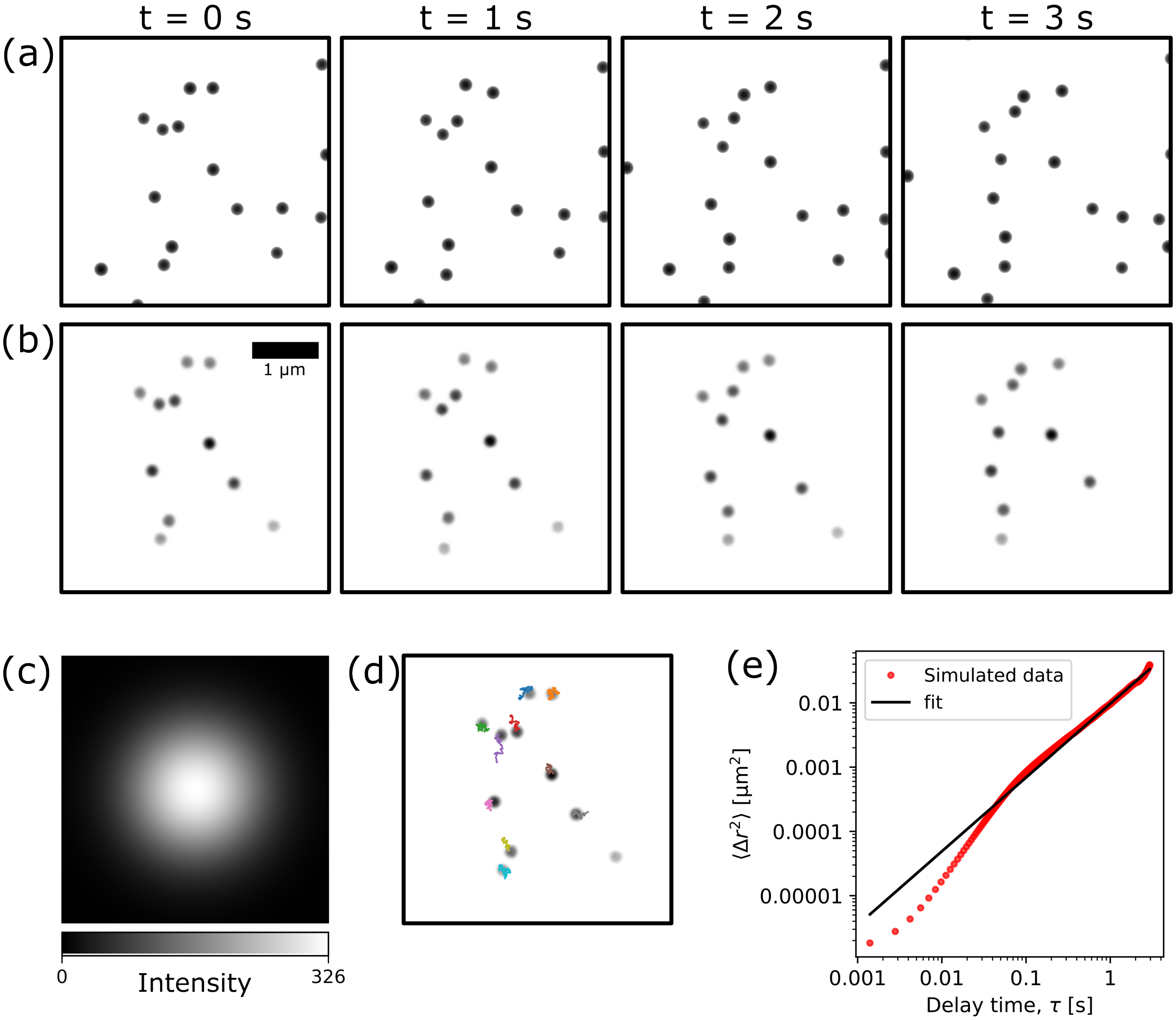

Figure S10: CXDI results from a simulation of 200 nm Au nanoparticles undergoing Brownian motion. (a) The simulated object amplitude at representative time points, and (b) the corresponding reconstructed amplitudes. (c) The simulated intensity of the probe function. All images have been cropped to a size of 4 × 4 μm^2, where the scalebar which applies to all images is shown in the first panel in (b). (d) The single particle tracking results performed after alignment of the time-series, where different nanoparticle trajectories are indicated by different colors. (e) A plot of the mean-squared displacement obtained from the single particle tracking results, where the calculated diffusion coefficient from the fit is $D = 2,441 \pm 772$ nm^2/s.

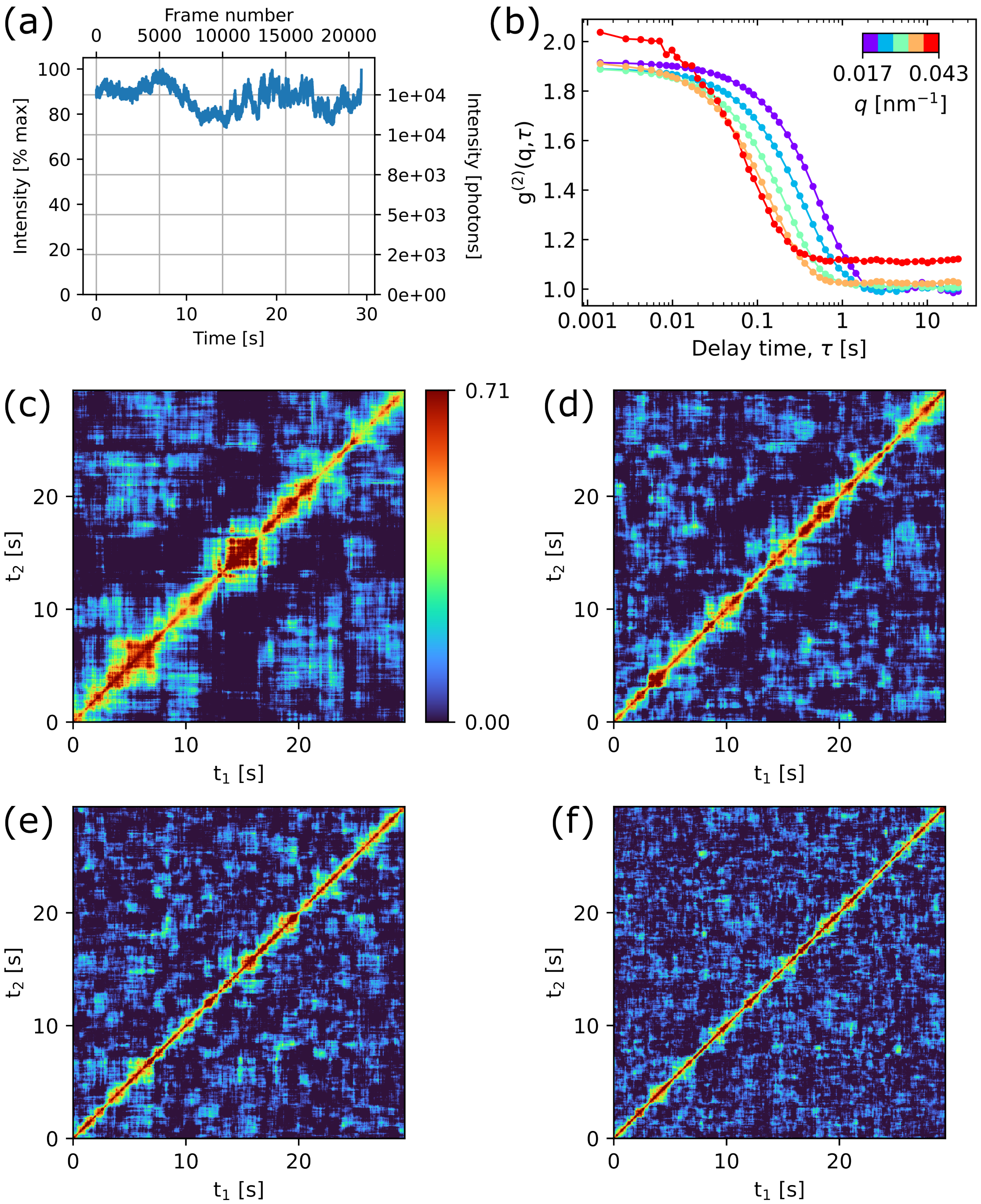

Figure S11: XPCS analysis performed on the simulated time-series data at 293 K. (a) Plot of the scattered intensity over the whole time-series. (b) Calculated $g^{(2)}(q, \tau)$ functions as a function of delay time, where the color represents different q-partitions used for the analysis. The q-partitions are spaced with equal dq/q steps. (c-f) Two-time correlation functions calculated at q-partitions of (c) 0.011 nm^{-1}, (d) 0.014 nm^{-1}, (e) 0.017 nm^{-1}, and (f) 0.022 nm^{-1}. All two-time correlations are plotted on the same color scale.

there is only Brownian diffusion, the fluctuating correlations in the maps are related to the number fluctuations and not any additional or more complex physical processes occurring within the system.

References

(1) Gold Nanoparticles - Nanopartz™. `https://www.nanopartz.com/`.

(2) Schulz, F.; Möller, J.; Lehmkühler, F.; Smith, A. J.; Vossmeyer, T.; Lange, H.; Grübel, G.; Schroer, M. A. Structure and Stability of PEG- and Mixed PEG-Layer-Coated Nanoparticles at High Particle Concentrations Studied In Situ by Small-Angle X-Ray Scattering. *Particle & Particle Systems Characterization* **2018**, *35*, 1700319.

(3) Lehmkühler, F.; Roseker, W.; Grübel, G. From Femtoseconds to Hours—Measuring Dynamics over 18 Orders of Magnitude with Coherent X-rays. *Applied Sciences* **2021**, *11*, 6179.

(4) Favre-Nicolin, V.; Girard, G.; Leake, S.; Carnis, J.; Chushkin, Y.; Kieffer, J.; Paleo, P.; Richard, M.-I. PyNX: high-performance computing toolkit for coherent X-ray imaging based on operators. *Journal of Applied Crystallography* **2020**, *53*, 1404–1413.

(5) Luke, D. R. Relaxed averaged alternating reflections for diffraction imaging. *Inverse Problems* **2005**, *21*, 37.

(6) Gerchberg, R.; Saxton, W. A Practical Algorithm for the Determination of Phase from Image and Diffraction Plane Pictures. *Optik (Stuttgart)* **1972**, *35*, 237–246.

(7) Marchesini, S.; He, H.; Chapman, H. N.; Hau-Riege, S. P.; Noy, A.; Howells, M. R.; Weierstall, U.; Spence, J. C. H. X-ray image reconstruction from a diffraction pattern alone. *Physical Review B* **2003**, *68*, 140101.

(8) Clark, J. N.; Huang, X.; Harder, R.; Robinson, I. K. High-resolution three-dimensional partially coherent diffraction imaging. *Nature Communications* **2012**, *3*, 993.

(9) Thevenaz, P.; Ruttimann, U.; Unser, M. A pyramid approach to subpixel registration based on intensity. *IEEE Transactions on Image Processing* **1998**, *7*, 27–41.

(10) Allan, D. B.; Caswell, T.; Keim, N. C.; van der Wel, C. M.; Verweij, R. W. soft-matter/trackpy: Trackpy v0.5.0. 2021; `https://zenodo.org/records/4682814`.

(11) Catipovic, M. A.; Tyler, P. M.; Trapani, J. G.; Carter, A. R. Improving the quantification of Brownian motion. *American Journal of Physics* **2013**, *81*, 485–491.

(12) Hinsley, G. Synchrotron X-ray Imaging with High Spatiotemporal Resolution. PhD Thesis, La Trobe, 2022.

(13) Cheng, N.-S. Formula for the Viscosity of a Glycerol-Water Mixture. *Industrial & Engineering Chemistry Research* **2008**, *47*, 3285–3288.

(14) Henke, B. L.; Gullikson, E. M.; Davis, J. C. X-Ray Interactions: Photoabsorption, Scattering, Transmission, and Reflection at E = 50-30,000 eV, Z = 1-92. *Atomic Data and Nuclear Data Tables* **1993**, *54*, 181–342.
